# A Dynamic Time Warping Based Algorithm to Evaluate Kinect-Enabled Home-Based Physical Rehabilitation Exercises for Older People

**DOI:** 10.3390/s19132882

**Published:** 2019-06-28

**Authors:** Xiaoqun Yu, Shuping Xiong

**Affiliations:** Department of Industrial and Systems Engineering, Korea Advanced Institute of Science and Technology (KAIST), Daejeon 34141, Korea

**Keywords:** aging, physical function, rehabilitation exercise, Kinect, dynamic time warping, automatic coaching, exergame

## Abstract

Older people face difficulty engaging in conventional rehabilitation exercises for improving physical functions over a long time period due to the passive nature of the conventional exercise, inconvenience, and cost. This study aims to develop and validate a dynamic time warping (DTW) based algorithm for assessing Kinect-enabled home-based physical rehabilitation exercises, in order to support auto-coaching in a virtual gaming environment. A DTW-based algorithm was first applied to compute motion similarity between two time series from an individual user and a virtual coach. We chose eight bone vectors of the human skeleton and body orientation as the input features and proposed a simple but innovative method to further convert the DTW distance to a meaningful performance score in terms of the percentage (0–100%), without training data and experience of experts. The effectiveness of the proposed algorithm was validated through a follow-up experiment with 21 subjects when playing a Tai Chi exergame. Results showed that the algorithm scores had a strong positive linear relationship (r = 0.86) with experts’ ratings and the calibrated algorithm scores were comparable to the gold standard. These findings suggested that the DTW-based algorithm could be effectively used for automatic performance evaluation of an individual when performing home-based rehabilitation exercises.

## 1. Introduction

The global healthcare system is under great pressure due to rapid population aging as well as a shortage of healthcare personnel and budget [1]. An increasing proportion of older people is facing serious challenges of impaired physical functions such as muscle strength, balance, and mobility [2]. All these negative changes result in difficulties for older people maintaining independence of daily living, which would further cause anxiety, low self-esteem, and decreased quality of life [3,4]. Epidemiological studies show that a low physical activity level is strongly correlated to functional decline of the elderly. Physical exercise is an effective way to counteract the age-related functional decline [5].

There is strong evidence that appropriate physical rehabilitation exercises can improve physical activity level and activities of daily living of older people [4,6,7]. Conventional exercise therapies for older people are generally conducted in a formal rehabilitation center or clinical setting, which requires direct supervision from a professional therapist. Even though conventional exercise therapies have been shown as effective to increase physical activities as well as improve motor functions and balance, they suffer from low rates of uptake and adherence [8,9] due to a lack of enjoyment, inconvenient transportation, and high cost [10]. For example, Kobayashi et al. [11] examined the effects of physical exercise on fall risks in older people living at home in a rural area. After a 13-week intervention, they reported that the motor functions of the elderly were improved. However, only 31.7% of the participants were fully adherent to the intervention program. Another study by Liu-Ambrose et al. [12] showed that the Otago Exercise Program (OEP) improved functional mobility and executive functioning in older people based on a 26-week experiment. However, they reported that only 19.4% of the participants finished the whole sessions of OEP and only 25.0% of participants just completed the half proportion of sessions in OEP.

Recently, with the emergence of more affordable motion sensors (such as Kinect and inertial sensors) and gaming technologies, the use of home-based exergame (exercise + gaming) for physical rehabilitation appears promising over passive conventional exercise therapies for the elderly with respect to long-term uptake [9,13,14,15]. Such home-based solutions can not only eliminate restrictions of distance and cost to the rehabilitation center, but also allow the elderly to flexibly adjust the training schedule and exercise intensity. Another big advantage of exergame is that it provides auto-coaching to compensate for the lack of global healthcare resources [16]. In this kind of auto-coaching system, there is usually a virtual avatar coach in the game environment. The coaching system will guide end users to follow virtual coach’s motions as well as track and evaluate their reproduced motions. However, because there is no real coach on site during the exercise, the scientific motion comparison for automatic performance evaluation is critical to guarantee the effectiveness of such a system.

Different algorithms have been proposed to assess exercise performance automatically. Lin et al. [17] developed a Kinect-based rehabilitation system utilizing a “seated Tai Chi” exercise to assist patients with movement disorders. They decomposed each form of Tai Chi into four poses and calculated the difference of joint angles between skeletons of standard pose and user’s actual pose, which were tracked by Kinect sensors. Muangmoon et al. [18] adopted a similar method to evaluate Thai dance performance. Even though this kind of motion comparison algorithm is easy to implement due to the simplification of extracting a few discrete poses from continuous motions, the quality of exercise could not be evaluated comprehensively. This is because the simplification has resulted in the loss of some essential exercise information such as motion continuity, pace, and intensity. Another group of researchers proposed to assess motion similarity based on the correlation coefficient between two time series of human motions from a virtual coach (standard motion) and a subject (actual motion) [19,20]. Even though this approach can evaluate the overall performance from the continuous motion data, it fails to take into account the typical time lag and speed variance between the older subject’s actual motion and the virtual coach’s standard motion during the exercise therapy. It is difficult for the older people to exactly follow the pace of standard motion due to the physical and cognitive impairments as well as the lack of exercise skills. Both previously mentioned disadvantages can be elegantly overcome by dynamic time warping.

Dynamic time warping (DTW) is a well-known approach for measuring time-series similarity, which minimizes the effects of time lag and distortion in the time axis due to speed variation [21,22]. DTW outputs the optimal alignment (least matching cost or cumulative distance) between two time series and it is widely applied in speech and gesture recognition [23]. Due to the advantages of DTW, some researchers applied it into the auto-coaching system for physical rehabilitation exercises at home. Saraee et al. [24] applied DTW into developing a remote monitoring system to evaluate home-based physical exercises. Since DTW itself could not generate a meaningful scaled score for performance evaluation, a physical therapist was required to remotely monitor the patient in real time through Webcam and determine whether a patient’s performance was acceptable or not. Semblantes et al. [25] and Saenz-de-Urturi and Garcia-Zapirain Soto [26] used DTW and binary classification to discriminate between correct and incorrect motions. Su et al. [27] utilized DTW and Kinect sensors to evaluate patients’ supplementary exercise at home for shoulder rehabilitation, when compared with the pre-recorded standard motion in the hospital. They integrated DTW with fuzzy logic to convert different DTW matching costs (DTW distances) to three performance levels: bad, good, and excellent. Wei et al. [28] applied DTW to measure the similarity between the motion data of the trainee and the standard motion of the trainer in dance teaching. They utilized existing training data and the experience of experts to determine three boundaries of DTW matching costs for categorizing individual performances into four levels: below average, average, good, and excellent. Most studies require large representative training data of each exercise to convert the matching cost from DTW to the final performance score. This task is resource-intensive and it is difficult to generalize the established conversion criteria for a specific exercise program to different ones. In addition, the final performance evaluation is categorical, and, thus, qualitative and not sensitive to recognize user’s gradual progress of exercise interventions. Chatzitofis et al. [29] and Mocanu et al. [30] developed home-based rehabilitation systems for heart health and physical activity. DTW was used to compute the quantitative performance score. However, how to convert the DTW matching cost to a quantitative score was not described and the quantitative scores were not validated with ground-truth ratings. Osgouei et al. [31] recently proposed an objective method for quantitative performance evaluation of rehabilitation exergames. Using shoulder abduction exercise as an example and two angle features (shoulder angle and arm angle), they presented how DTW was applied to compute the motion similarity between the unknown and reference trajectories of the human skeleton joints. A normalization approach with estimated lower and upper bounds for the DTW distance was utilized to further convert any DTW distance to an objective similarity score (0–100). The proposed method is promising since it does not require any training data. However, the proposed objective similarity score was not validated with physicians’ evaluation of the performances. In addition, it remains questionable whether the proposed method can be extended from simple repetitive exercises to complex whole-body exercises.

This study aims to develop and validate a DTW-based algorithm for a motion similarity evaluation, in order to support effective Kinect-enabled home-based virtual coaching. We proposed a simple but innovative method to directly convert the DTW matching cost to a meaningful performance score in terms of percentage (0–100%), without training data and experience of experts. We further validated the effectiveness of our algorithm through a follow-up experiment with human subjects performing the complex whole-body exercise (Tai Chi) instead of simple, repetitive exercises, which would show good generalization of our proposed method to different exercise programs. The developed algorithm is expected to provide a similar evaluation on user’s performance as domain experts, which could be very promising to apply into home-based physical rehabilitation exercises for better quality of life of the elderly.

## 2. Methods

### 2.1. Development of a DTW-Based Algorithm for Performance Evaluation

#### 2.1.1. 3D Pose Comparison

**Figure 1:**
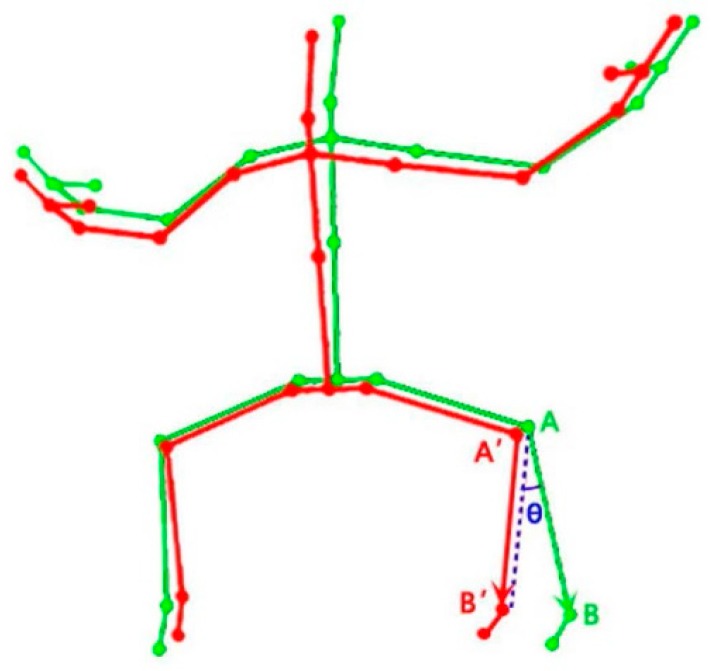
Angle difference (*Ɵ*) between corresponding bone vectors for trainer (green) and trainee (red), using the left lower leg as an example.

Human motion is the coordinated movement of different body parts, and motion data from the Kinect sensor could be seen as a sequence of frames that comprise 3D coordinates of joint positions of the human skeleton and each bone determined by two connected joints could be seen as a 3D vector in the space. In order to assess the similarity between trainer’s motion (coach) and trainee’s motion (end user), the first step is to quantify the difference between these two 3D poses at a given frame. In this study, we chose the sum of the angle difference between all corresponding bone vectors of the two 3D poses as the distance measure. Eight major bones of the human skeleton (the upper and lower arms, and the upper and lower legs) were chosen for motion comparison since most human motions during the rehabilitation exercises involve the coordination of upper and lower limb movements. The angle difference (*Ɵ*) between two corresponding bone vectors of trainer and trainee is illustrated in Figure 1, which can be calculated by the law of cosine (see Equation (1)).

(1) $$\theta \;=\;\mathrm{acos}\left(\frac{\vec{AB}\cdot \vec{A^{\prime }B^{\prime }}}{\left|\vec{AB}\right|\cdot \left|\vec{A^{\prime }B^{\prime }}\right|}\right)$$

#### 2.1.2. Motion Comparison

Given two sequences of motion data X and Y, each frame of motion data is a 3D pose. We applied DTW to find the optimal matching between the trainer’s motion and the trainee’s motion while minimizing the effects of shifting and distortion in time [32]. Since we chose eight bone vectors for a motion comparison and the motion of each bone constitutes one dimensional time-series data, both trainer and trainee’s data have eight dimensions. The matrix of each dataset has dimension 8-by-*n*, where *n* is the total frames of motion data. To explain how DTW works, let us start with the one-dimensional case. The motion of a body part can be denoted as *S* = (*s*_1_, *s*_2_, …, *s_n_*) and *T* = (*t*_1_, *t*_2_, …, *t_m_*), which correspond to the trainer’s motion and trainee’s motion, respectively. The element in *S* and *T* is a normalized bone vector of that body part in a certain 3D coordinate system. To compare the similarity of sequence *S* and *T* by DTW, an *n*-by-*m* cost matrix is constructed where the (*i*th, *j*th) element denoted as *C*(*s_i_*, *t_j_*) is the angle difference between *s_i_* and *t_j_* (See Equation (1)). A warping path denoted as *P* defines an alignment between *S* and *T* in the cost matrix, which should satisfy three conditions: boundary condition, monotonicity condition, and step size condition [33]. There could be multiple feasible warping paths in the cost matrix and the total matching cost of one warping path *P* between *S* and *T* is defined by the equation below.

(2) $$c_{p}\left(S,\;T\right)\;=\;\sum_{k\;=\;1}^{s}C\left(s_{ik},\;t_{jk}\right),$$

where *s* is the length of the warping path *P*.

The goal of DTW is to find the optimal warping path, which has the minimal cumulative distance among all the possible warping paths. The DTW distance *DTW(S*, *T)* is defined as the total matching cost of the optimal warping path. In order to find the optimal warping path, a dynamic programming method is used. The recursive equation is given by the equation below.

(3) $$D\left(i,j\right)\;=\;min\left\{D\left(i-1,\;j-1\right),\;D\left(i-1,\;j\right),D\left(i,\;j-1\right)\right\}+C\left(s_{i},t_{j}\right),$$

where 1 < *i* < *n* and 1 < *j* < *m*. *D*(*i*, *j*) represents the matching cost between standard data (*S*) and testing data (*T*) from (1, 1) to (*i*, *j*).

DTW can be generalized from the one-dimensional case to a multi-dimensional case [34]. For a multidimensional case, *s_i_* and *t_j_* are not single bone vector but multiple bone vectors, which represent whole body motion. Multi-dimensional *DTW*(*S*, *T*) is calculated in a similar way as the one-dimensional case, except that we need to redefine *C*(*s_i_*, *t_j_*) as the sum of angle difference among all the dimensions. The output of DTW is the matching cost associated with the cumulative distance along the shortest warping path. Therefore, the lower the matching cost is, the closer the two motion sequences are and the better the motion performance is. In order to quantitatively assess motion performance of a trainee, we further convert the DTW distance (matching cost) to a meaningful performance score in terms of the percentage (0–100%) using the following equation.

(4) $$Performance\;score\;=\;\frac{\sum_{k\;=\;1}^{s}\left[1-\frac{C\left(s_{ik},\;t_{jk}\right)}{90\times 8}\right]}{s}\;=\;1-\frac{DTW\left(S,\;T\right)}{90\times 8\times s},$$

where *s* is the length of optimal warping path, 8 stands for eight bone vectors selected for motion evaluation, and *C*(*s_ik_*, *t_jk_*) is the element of optimal warping path in the cost matrix, which is the summation of angle differences for eight bone vectors, and DTW distance-*DTW*(*S*, *T*) is a summation of elements (*C*(*s_ik_*, *t_jk_*)) along the optimal path. We assume the angle difference between two corresponding bone vectors is within 90 degrees based on an earlier study [18], which results in the maximum *DTW*(*S*, *T*) along the optimal path, which would be 90 × 8 × s. Because the output distance (*DTW*(*S*, *T*)) is a measure of dissimilarity between the two motion time series (the longer the distance, the greater the deviation), the last part of Equation (4) would be a percentage score (0–100%) to measure the level of similarity between the trainee’s motion and trainer’s motion.

#### 2.1.3. Body Orientation Offset

Calculation of bone vectors for both trainee’s motion and trainer’s motion based on the world coordinate system could be problematic if the trainee is not oriented as exactly as the trainer. The difference (error) in body orientation would be directly transferred to all eight bone vectors. In order to solve this problem, instead of using the joint position based on the world coordinate system, we calculate the joint position data based on a local coordinate system of the human model, which would be updated in real time. Establishment of the local coordinate system was adopted from Unity3D Mecanim system [35]. The up vector is defined as middle of left/right upper arm and middle of left/right upper leg. The left vector is an average upper body left (a vector defined by the left upper arm and the right upper arm) and lower body left (a vector defined by the left upper leg and the right upper leg). The forward vector is the cross product of the up vector and the left vector. In order to make these three vectors orthogonal to each other, the final left vector is the cross product of the forward vector and the up vector. Then the up vector is aligned to a normal vector of a ground plane as the y-axis. Hence, the final left vector and forward vector would be finalized as the x-axis and z-axis, respectively, based on the rotation matrix. The main purpose of this step is to guarantee that the x-axis and the z-axis would always be in the ground plane. The origin of this local coordinate system is the projection of center of mass on the ground.

**Figure 2:**
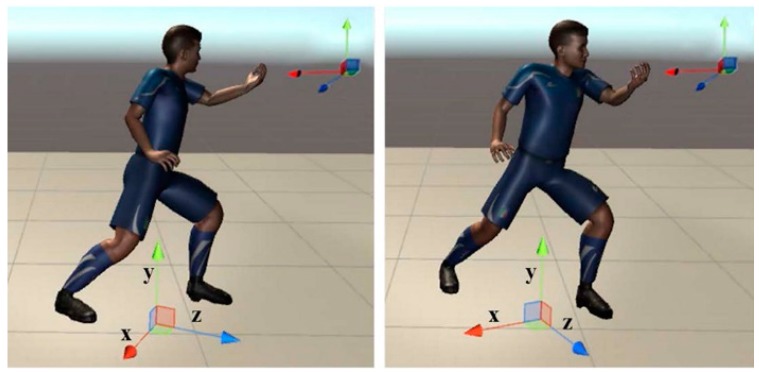
Illustration of motion similarity evaluation under the local coordinate system of the human model (bottom) and the world coordinate system (at the top right corner).

As shown in Figure 2, the only difference between motions of two avatars is the body orientation (see z-axis). Under the local coordinate system, all the bone vectors between two avatars are exactly the same. While under the world coordinate system, there are clear angle differences among all corresponding bone vectors of two avatars, which are caused by the different body orientations.

Calculation of angular differences based on the local coordinate system of the human model can remove the accumulative errors on eight bone vectors caused by different body orientations. However, the difference in body orientation between trainee and trainer should also be considered in this case. Hence, we added one more dimension-body orientation into the evaluation of motion similarity. Lastly, we used nine dimensions to calculate the motion similarity: eight bone vectors under the local coordinate system of the human model and the forward vector of the body, which reflects the body (mainly trunk) orientation (z-axis in Figure 2). Then the final performance score can be updated by Equation (5).

(5) $$Final\;performance\;score\;=\;\frac{\sum_{k\;=\;1}^{s}\left[1-\frac{C\left(s_{ik},\;t_{jk}\right)}{90\times 9}\right]}{s}\;=\;1-\frac{DTW\left(S,\;T\right)}{90\times 9\times s},$$

where all the notations are the same as in Equation (4) and where 9 stands for nine dimensions.

### 2.2. Validation of the Developed DTW-Based Algorithm

In order to validate the developed algorithm, we performed a follow-up experiment where the final performance scores from the algorithm were compared with ratings given by the domain experts. An 8-form Tai Chi exercise was selected to evaluate the proposed algorithm.

#### 2.2.1. Experimental Participants

Twenty-one middle-aged and older subjects (age: 55.2 ± 4.2 years, height: 166.1 ± 7.9 cm, weight: 65.36 ± 8.3 kg) from a local Tai Chi academy participated in the algorithm validation experiment. For the sake of convenience, the experiment was conducted in the same Tai Chi academy instead of each subject’s home. All the subjects were in healthy conditions and without musculoskeletal diseases or injuries that may affect their Tai Chi performance. Prior to the participation of the experiment, each subject signed informed consent on the experiment protocol, which was approved by the KAIST Institutional Review Board (IRB-18-070).

#### 2.2.2. Experimental Setup and Procedure

**Figure 3:**
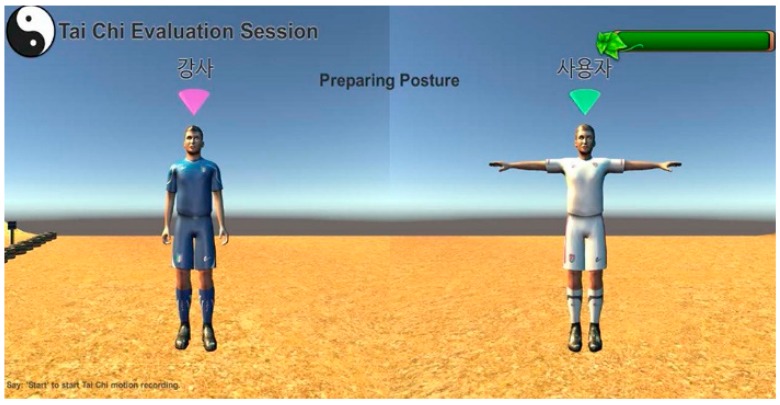
Main scene of the Tai Chi exergame (left: trainer avatar, right: trainee avatar).

The Tai Chi exergame was developed with Kinect V2 sensor in Unity3D platform (Unity 5.5.2f1) by C# for the real-world application. The main scene of the exergame is implemented with two avatars: virtual trainer and trainee (Figure 3). The avatar-based rendering of motion preserves the privacy of the user, which is critical for the healthcare systems. The motion of the trainee avatar is updated by the real motion of the user. User’s motion is captured by Kinect V2 (Microsoft Corp, Redmond, WA, USA) and mapped to the trainee avatar using the Kinect V2 asset for Unity3D [36]. The motion of the trainer avatar is retargeted by the pre-recorded standard motion from a certified Tai Chi instructor (Master Level).

**Figure 4:**
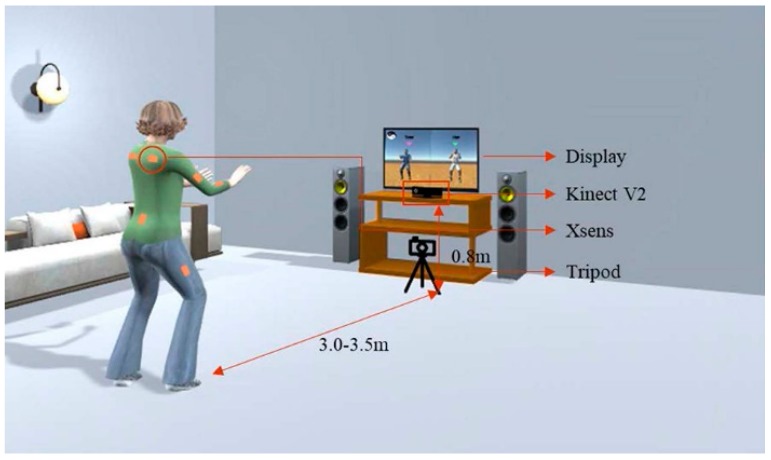
Experimental setup for Tai Chi motion evaluation.

The whole experimental setting is illustrated in Figure 4. Both Kinect V2 and Xsens motion capture system were used to capture each subject’s motion. The Kinect sensor was placed at a height of 0.8 m. Since subjects should stretch their arms frequently because of the characteristics of Tai Chi motion, subjects were instructed to stand around 3.0–3.5 m away from the Kinect sensor. Considering that some Tai Chi motion with body rotation may not be well captured by the Kinect due to the self-occlusion [37], a wearable inertial sensor-based motion capture system-Xsens MVN BIOMECH (Xsens Technologies B.V., Enschede, The Netherlands) was also utilized to obtain high-quality motion data for the algorithm validation [38,39].

A smart phone, supported by a tripod, was used to record each subject’s motion when he/she was playing the Tai Chi exergame. The motion videos were distributed to three Tai Chi experts for independent performance evaluation. Each expert was provided a 10-cm visual analog scale (VAS) [40] and asked to place a vertical mark on the scale to indicate the performance level of motion for each subject. The anchor statements for VAS in this study are “cannot follow Tai Chi at all” (score of 0) on the left and “master level with standard Tai Chi motion” (score of 100) on the right. The raw scale score is then converted to a 0–100 scale.

#### 2.2.3. Statistical Analysis

The intraclass correlation coefficient (ICC) was used to check the inter-rater reliability of experts’ subjective ratings [41]. Good consistency and agreement among different experts are the prerequisite to consider experts’ rating as the gold standard for validating the developed DTW-based algorithm. ICC is a widely used reliability index and the general guideline of ICC is as follows: ICC < 0.5, poor reliability, 0.5 < ICC < 0.75, moderate reliability, 0.75 < ICC < 0.9, good reliability, and ICC > 0.9, excellent reliability [42].

More importantly, final performance scores from the developed algorithm were compared with the experts’ ratings (as a gold standard). The Pearson correlation coefficient (r) between final performance scores from the developed algorithm and those from experts was calculated to assess the strength of a linear relationship between those two evaluation methods. In addition, linear regression was used to calibrate performance scores from the algorithm so that the scores from two evaluation methods could be consistent. Differences between algorithm scores after calibration and experts’ ratings were analyzed. The SPSS statistical package version 20 (IBM Corp., Armonk, NY, USA) was used for statistical analysis.

## 3. Results

### 3.1. Inter-Rater Reliability of Experts’ Ratings

**Figure 5:**
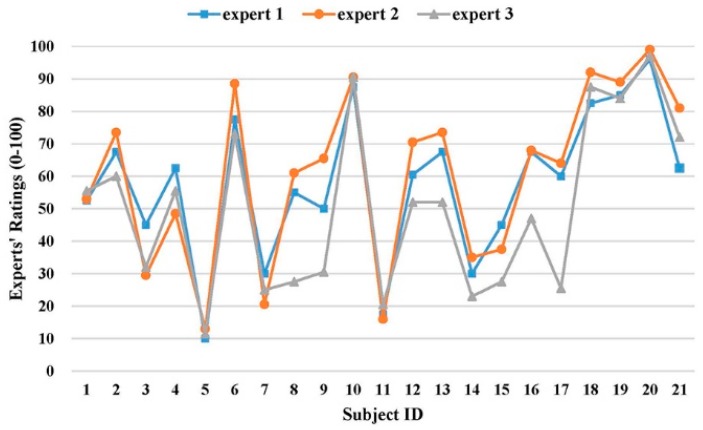
Experts’ ratings on motion performance of 21 subjects.

Figure 5 shows the subjective ratings of 21 subjects from three independent experts. The ICC value for three experts was 0.861 (95% CI: 0.688~0.942), which was within the range of 0.75 to 0.9 [41,42], which indicates the experts’ ratings were consistent and with good inter-rater reliability.

### 3.2. Evaluation Comparison between Experts and the Developed Algorithm

**Figure 6:**
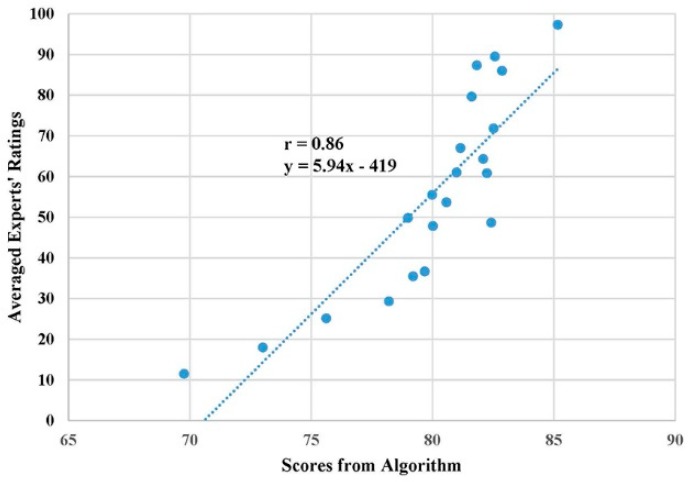
Linear relationship between algorithm scores and experts’ ratings.

**Figure 7:**
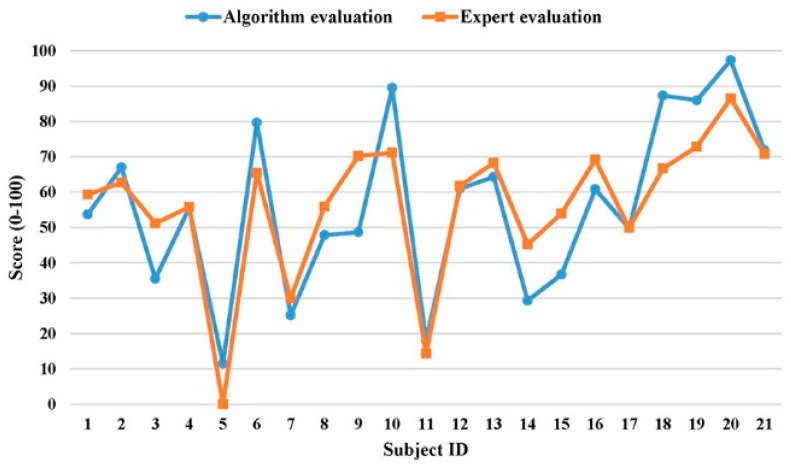
Comparison between algorithm evaluation (after calibration) and experts’ evaluation.

The subjective ratings from three experts were averaged and regarded as the gold standard to validate the developed DTW-based algorithm. Figure 6 shows the scatter plot of the algorithm’s final performance scores and averaged experts’ ratings for 21 subjects. The Pearson correlation coefficient (r) was 0.86 (*t* = 7.45, *p* < 0.001), which indicates a strong positive linear relationship between scores from those two evaluation methods. Additional analysis on performance scores between two evaluation methods showed that, under many circumstances, the algorithm would overestimate the performance when compared with the experts’ rating. Therefore, we calibrated performance scores of the algorithm using the fitted equation from linear regression (see Figure 6) so that the algorithm would generate similar evaluation scores as the domain experts for practical applications. Figure 7 further demonstrates the calibrated performance scores from the algorithm were comparable to experts’ ratings. The score difference between two evaluation methods had a mean of 9.5 and a standard deviation of 7.0 (Maximum: 21.6, Minimum: 0.1).

## 4. Discussion

We developed a DTW-based algorithm for assessing motion similarity between an individual user and a virtual coach. DTW was designed to handle local changes in timing (due to speed variations) and, therefore, desirable for evaluating rehabilitation exercises for the elderly self-care at home. The effectiveness of the algorithm was validated through a follow-up experiment. In the validation experiment, the Tai Chi exercise was chosen as the representative physical exercise to verify the proposed algorithm due to two major reasons. First, the effectiveness of Tai Chi exercise for improving physical functions has been proven by many previous studies [43,44]. Second, Tai Chi exercise is a complex and whole-body motion. If the algorithm could perform well in terms of evaluating Tai Chi motion, it should be generalizable to other simpler rehabilitation exercises.

Inter-rater reliability analysis revealed that the reliability level of experts’ ratings was “good” (0.75 < ICC = 0.861 < 0.90). However, the 95% confidence interval of ICC was wide (0.688–0.942), which indicates that, in the worst case, the reliability level was just “acceptable” (0.5 < ICC = 0.688 < 0.75). The wide confidence interval warned that, even though the overall agreement were high among three experts, there were non-negligible disagreement on their ratings [45]. Paired t-tests also confirmed the significant difference on performance ratings between the third expert and the other two experts (Figure 5). The inconsistency on subjective ratings from three experts highlighted the potential benefits of applying our developed algorithm to assess the exercise performance automatically and objectively.

Strong linear relationship (r = 0.86) between the algorithm score and experts’ evaluation (gold standard) implied the developed algorithm was sensitive in terms of recognizing the performance levels from different subjects as the domain experts. Unexpectedly, a detailed analysis revealed that the algorithm score was significantly higher than the experts’ rating. This could be mainly due to different baselines for two evaluation methods. The algorithm evaluation was purely based on the sum of angle differences among nine corresponding body vectors and the subjects with all angle differences at 90 degrees were considered as the worst (performance score = 0). Since even the subjects rated by the experts as the worst in terms of motion performance had most of the angle differences within 45 degrees, the algorithm evaluation would overestimate the subject’s performance score due to the ceiling effect [46]. To reduce this overestimation and enable our algorithm to provide similar scores as the domain experts, the linear regression equation (Figure 6) was applied to calibrate the algorithm score. The experimental results showed that the calibrated algorithm scores were comparable to the experts’ ratings. Taken together, these findings demonstrated that, even though the developed DTW-based algorithm could be a good evaluation tool to rank the exercise performance among different subjects objectively, the algorithm score should be calibrated by experts’ ratings on a small number of representative subjects. In this way, the good consistency between algorithm evaluation and experts’ evaluation can be achieved for the practical applications.

Earlier studies used binary classification as well as three-point and four-point Likert scales to obtain experts’ ratings for validating their algorithms [24,25,26,27,28]. This kind of validation is rough and likely results in inflated validation accuracy because of the wide performance range between two consecutive points, especially for binary classification and a 3-point Likert scale. To the best of our knowledge, there was only one reported study, which also used 0–100 score as the experts’ rating as we did to validate the developed algorithm [21]. However, the highest correlation coefficient between their DTW-based algorithm score and expert’s rating was 0.64, which was much lower than ours (r = 0.86). The improved performance from our study could be related to the selection of different motion features. Instead of using simple joint angles as Capecci et al. [21], 3D bone vectors of human skeletons were chosen in our study for better conservation of spatial information of the motion because joint angles could not define spatial information of two bones connected by the same joint. In addition, since there always exist theoretical upper and lower bounds (180 and 0 degrees) for any angle difference between two corresponding bone vectors, converting the DTW matching cost to a final percentage score is straightforward and reasonable in our study. It does not require training data and experience of experts. In this study, we assumed the upper bound was 90 degrees instead of 180 degrees based on an earlier study [18] and our practical exercise scenario.

It is worthwhile to mention that elimination of the confounding effect caused by body orientation offset is a major challenge for the algorithm development. In fact, both bone vector-based and joint position-based algorithms are very sensitive to the body orientation especially when evaluating complex whole-body exercises with rotational motions. Chua et al. [47] also pointed out this issue when they evaluated Tai Chi motion. We calculated the joint positions and bone vectors based on the local coordinate system of the human model instead of the world coordinate system in real-time, which can get rid of the error induced by the body orientation offset during the entire exercise. The compensation of body orientation offset had practical meaning for the elderly because they might not be able to orient themselves precisely as the standard virtual coach during the rehabilitation exercise.

**Figure 8:**
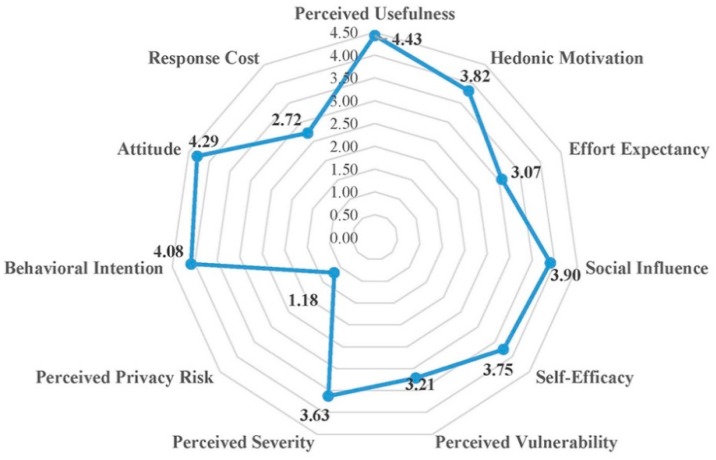
Results of the user acceptance questionnaire for Tai Chi exergaming prototype system. Remarks: (1) Scores from all older participants were averaged for each construct. (2) Except perceived privacy risk and response cost, all constructs are positively associated with user’s intention to adopt the system.

In order to further examine the use and acceptance of exergaming technology for home-based physical rehabilitation by the primary target users (older people), we applied the technology acceptance model [48,49,50,51] and designed a questionnaire with 11 constructs (Appendix A) to evaluate user acceptance of our developed Tai Chi exergaming prototype system (Figure 3). Forty-one older adults (age 77.3 ± 5.4 years, height 159.3 ± 8.5 cm, weight 59.0 ± 9.5 kg) from a local senior welfare center participated in this survey. They were asked to try the prototype system and play the Tai Chi exergame before giving their questionnaire responses in a five-point Likert scale (1 corresponding to “strongly disagree” and 5 corresponding to “strongly agree”). Figure 8 presents a summary of their responses. The results showed that the older people perceived relatively high vulnerability (3.21 out of 5) and severity (3.63) in terms of difficulties in self-care and independent living, and they had high intentions (Behavior intention = 4.08) to use our system in the future. They thought our system was very useful (Perceived usefulness = 4.43), positive (Attitude = 4.29), entertaining (Hedonic motivation = 3.82) and having low privacy risk (Perceived privacy risk = 1.18). Interestingly, even though the older people were somewhat confident in their capabilities to use this system for improving their health conditions (Self-efficacy = 3.75), the expected effort (3.07) and response cost (2.72) were considerably high. Taken all together, these findings implied that our developed Tai Chi exergaming prototype system is useful for the older people performing home-based physical rehabilitation exercises. However, the prototype system needs be improved to make it easy to use and cost-effective. We collected some valuable feedback from the participants to improve our prototype system, which mainly includes the following: (1) Audio effects should be added to make the exergame more entertaining and enjoyable. (2) The standard pace of Tai Chi exergame should slow down and be adjustable by each individual. (3) The size of avatar should be enlarged to be seen clearly and timely feedback for problematic motions should be provided, and (4) social networking functions (such as sharing exergaming performance score with friends) should be further developed.

There were several limitations in the current study. First, the conversion from DTW distance to a percentage score (0–100%) is based on the assumption that the maximum angle difference between two corresponding bone vectors is 90 degrees. Even though this assumption works fine for most body parts, the exact value of 90 is not always appropriate. Second, we focused on motion correctness for the performance evaluation in this study. The rhythm mismatch was not yet considered in the overall performance evaluation [28]. In addition, detailed feedback for problematic motions from certain body parts should be provided in the future study to timely inform the older individual for further improvements in rehabilitation exercises. Third, even though the primary target users for our developed Tai Chi exergaming system are older adults, 21 participants for validating DTW-based algorithm included both middle-aged and older adults, in order to cover a wide range of Tai Chi proficiency levels under practical constraints. Our next step will be to refine the developed prototype system and test it with a large number of older adults at the home environment for verifying practicality of the system. Last but not least, a single Kinect sensor often generates poor skeleton tracking performance for some rotational motions during rehabilitation exercises due to self-occlusion and limited sensing range [37]. Further research on combining data from multiple Kinect sensors to achieve more accurate and robust skeleton tracking performance is needed.

## 5. Conclusions

We developed a DTW-based algorithm to automatically evaluate user’s performance during physical rehabilitation exercises. We chose eight bones vectors of the human skeleton and body orientation as the input features and proposed a simple but innovative method to further convert the DTW matching cost to a meaningful performance score in terms of percentage (0–100%), without training data and experience of experts. The effectiveness of the proposed algorithm was tested through a follow-up experiment with 21 subjects when playing a complex whole-body exercise (Tai Chi) instead of simple repetitive exercises. Results showed that the algorithm scores had a strong positive linear relationship (r = 0.86) with experts’ ratings and the calibrated algorithm scores were comparable to the gold standard. These findings suggested that our algorithm could be effectively used for automatic performance evaluation of an older individual when performing home-based physical rehabilitation exercises.

## Appendix A

**Table A1:** Questionnaire for user acceptance evaluation.

Construct ID	Questions	Strongly Disagree	Disagree	Undecided	Agree	Strongly Agree
1	Perceived Usefulness (PU)					
	PU1: Using this system will help improve my health quality.	1	2	3	4	5
	PU2: Using this system will make me more effective in my life.	1	2	3	4	5
	PU3: Overall, I find this system to be useful in my life.	1	2	3	4	5
2	Hedonic Motivation (HM)					
	HM1: Using this system is fun.	1	2	3	4	5
	HM2: Using this system is enjoyable.	1	2	3	4	5
	HM3: Using this system is entertaining.	1	2	3	4	5
3	Effort Expectancy (EE)					
	EE1: Learning how to use this system is easy for me.	1	2	3	4	5
	EE2: I find this system easy to use.	1	2	3	4	5
	EE3: It is easy for me to become skillful at using this system.	1	2	3	4	5
4	Social Influence (SI)					
	SI1: People who are important to me would think that I should use this system.	1	2	3	4	5
	SI2: People who influence me would think that I should use this system.	1	2	3	4	5
	SI3: People whose opinion are valued to me would prefer that I use this system.	1	2	3	4	5
5	Self-Efficacy (SE)					
	SE1: It is easy for me to improve my health conditions by using this system.	1	2	3	4	5
	SE2: I have the capability to use this system to improve my health condition.	1	2	3	4	5
	SE3: I am able to use this system to improve my health condition without much effort.	1	2	3	4	5
6	Perceived Vulnerability (PV): having little knowledge about self-care and improving personal healthcare; suffering falls.					
	PV1: I am at the risk of suffering from the stated problems.	1	2	3	4	5
	PV2: It is likely that I will suffer the stated problems.	1	2	3	4	5
	PV3: It is possible for me to suffer the stated problems.	1	2	3	4	5
7	Perceived Severity (PS): having little knowledge about self-care and improving personal healthcare; suffering falls.					
	PS1: If I suffered the stated problems, it would be severe.	1	2	3	4	5
	PS2: If I suffered the stated problems, it would be serious.	1	2	3	4	5
	PS3: If I suffered the stated problems, it would be significant.	1	2	3	4	5
8	Perceived Privacy Risk (PPR)					
	PPR1: It would be at risk to disclose my personal health information to the doctor.	1	2	3	4	5
	PPR2: There would be high potential for loss associated with disclosing my personal health information to the doctor.	1	2	3	4	5
	PPR3: There would be too much uncertainty associated with giving my personal health information to the doctor.	1	2	3	4	5
9	Behavioral Intention (BI)					
	BI1: I intend to use this system in the future.	1	2	3	4	5
	BI2: I intend to use this system at every opportunity in the future.	1	2	3	4	5
	BI3: I plan to increase my use of this system in the future.	1	2	3	4	5
10	Attitude (AT)					
	AT1: Using this system is a good idea.	1	2	3	4	5
	AT2: Using this system is a wise idea.	1	2	3	4	5
	AT3: I like the idea of using this system.	1	2	3	4	5
11	Response Cost (RC)					
	RC1: This system is expensive to purchase (the price of Kinect is $150).	1	2	3	4	5
	RC2: I have to spend effort on learning how to use this system.	1	2	3	4	5
	RC3: Using this system will change my life style.	1	2	3	4	5

## References

[1] World Health Organization Ageing and Health. https://www.who.int/news-room/fact-sheets/detail/ageing-and-health.

[2] Weening-Dijksterhuis E., de Greef M.H., Scherder E.J., Slaets J.P., van der Schans C.P. (2011). Frail institutionalized older persons: A comprehensive review on physical exercise, physical fitness, activities of daily living, and quality-of-life. Am. J. Phys. Med. Rehabil..

[3] Windle G., Hughes D., Linck P., Russell I., Woods B. (2010). Is exercise effective in promoting mental well-being in older age? A systematic review. Aging Ment. Health.

[4] Frändin K., Grönstedt H., Helbostad J.L., Bergland A., Andresen M., Puggaard L., Harms-Ringdahl K., Granbo R., Hellström K. (2016). Long-term effects of individually tailored physical training and activity on physical function, well-being and cognition in Scandinavian nursing home residents: A randomized controlled trial. Gerontology.

[5] Brach J.S., FitzGerald S., Newman A.B., Kelsey S., Kuller L., VanSwearingen J.M., Kriska A.M. (2003). Physical activity and functional status in community-dwelling older women: A 14-year prospective study. Arch. Intern. Med..

[6] Rydwik E., Frändin K., Akner G. (2010). Effects of a physical training and nutritional intervention program in frail elderly people regarding habitual physical activity level and activities of daily living—A randomized controlled pilot study. Arch. Gerontol. Geriatr..

[7] Organization W.H. (2007). A guide for population-based approaches to increasing levels of physical activity. Implementation of the WHO Global Strategy on Diet, Physical Activity and Health.

[8] Lin S.F., Lee J.W., Modeste N., Johnson E.G. (2012). Attitudes and beliefs predicting Taiwanese older adults’ intentions to attend strength and balance training programs. J. Appl. Gerontol..

[9] Choi S.D., Guo L., Kang D., Xiong S. (2017). Exergame technology and interactive interventions for elderly fall prevention: A systematic literature review. Appl. Ergon..

[10] Yardley L., Bishop F.L., Beyer N., Hauer K., Kempen G.I., Piot-Ziegler C., Todd C.J., Cuttelod T., Horne M., Lanta K. (2006). Older people’s views of falls-prevention interventions in six European countries. Gerontologist.

[11] Kobayashi R., Nakadaira H., Ishigami K., Muto K., Anesaki S., Yamamoto M. (2006). Effects of physical exercise on fall risk factors in elderly at home in intervention trial. Environ. Health Prev. Med..

[12] Liu-Ambrose T., Donaldson M.G., Ahamed Y., Graf P., Cook W.L., Close J., Lord S.R., Khan K.M. (2008). Otago home-based strength and balance retraining improves executive functioning in older fallers: A randomized controlled trial. J. Am. Geriatr. Soc..

[13] Uzor S., Baillie L. Investigating the long-term use of exergames in the home with elderly fallers. Proceedings of the SIGCHI Conference on Human Factors in Computing Systems.

[14] Arlati S., Colombo V., Spoladore D., Greci L., Pedroli E., Serino S., Cipresso P., Goulene K., Stramba-Badiale M., Riva G. (2019). A Social Virtual Reality-Based Application for the Physical and Cognitive Training of the Elderly at Home. Sensors.

[15] Fortino G., Gravina R. (2014). A cloud-assisted wearable system for physical rehabilitation. ICTs for Improving Patients Rehabilitation Research Techniques.

[16] Ofli F., Kurillo G., Obdržálek Š., Bajcsy R., Jimison H.B., Pavel M. (2016). Design and evaluation of an interactive exercise coaching system for older adults: Lessons learned. IEEE J. Biomed. Health Inform..

[17] Lin T.Y., Hsieh C.H., Lee J.D. A kinect-based system for physical rehabilitation: Utilizing Tai Chi exercises to improve movement disorders in patients with balance ability. Proceedings of the Asia Modelling Symposium 2013: 7th Asia International Conference on Mathematical Modelling and Computer Simulation.

[18] Muangmoon O.-O., Sureephong P., Tabia K. (2017). Dance Training Tool Using Kinect-Based Skeleton Tracking and Evaluating Dancer’s Performance. International Conference on Industrial, Engineering and other Applications of Applied Intelligent Systems.

[19] Alexiadis D.S., Kelly P., Daras P., O’Connor N.E., Boubekeur T., Moussa M.B. Evaluating a dancer’s performance using kinect-based skeleton tracking. Proceedings of the 19th ACM International Conference on Multimedia.

[20] Ren W., Pu F., Fan X., Li S., Sun L., Li D., Wang Y., Fan Y. (2016). Kinect-Based Skeleton-Matching Feedback for Motor Rehabilitation: Transient Performance Effect of Shoulder training. J. Mech. Med. Biol..

[21] Capecci M., Ceravolo M.G., Ferracuti F., Iarlori S., Kyrki V., Longhi S., Romeo L., Verdini F. Physical rehabilitation exercises assessment based on hidden semi-markov model by kinect v2. Proceedings of the 2016 IEEE-EMBS International Conference on Biomedical and Health Informatics (BHI).

[22] Rybarczyk Y., Deters J.K., Gonzalo A.A., Esparza D., Gonzalez M., Villarreal S., Nunes I.L. (2017). Recognition of physiotherapeutic exercises through DTW and low-cost vision-based motion capture. International Conference on Applied Human Factors and Ergonomics.

[23] Müller M., Röder T. (2006). Motion templates for automatic classification and retrieval of motion capture data. Proceedings of the 2006 ACM SIGGRAPH/Eurographics Symposium on Computer Animation.

[24] Saraee E., Singh S., Hendron K., Zheng M., Joshi A., Ellis T., Betke M. ExerciseCheck: Remote monitoring and evaluation platform for home based physical therapy. Proceedings of the 10th International Conference on PErvasive Technologies Related to Assistive Environments.

[25] Semblantes P.A., Andaluz V.H., Lagla J., Chicaiza F.A., Acurio A. (2018). Visual feedback framework for rehabilitation of stroke patients. Inform. Med. Unlocked.

[26] Saenz-de-Urturi Z., Garcia-Zapirain Soto B. (2016). Kinect-based virtual game for the elderly that detects incorrect body postures in real time. Sensors.

[27] Su C.-J., Chiang C.-Y., Huang J.-Y. (2014). Kinect-enabled home-based rehabilitation system using Dynamic Time Warping and fuzzy logic. Appl. Soft Comput..

[28] Wei Y., Yan H., Bie R., Wang S., Sun L. (2014). Performance monitoring and evaluation in dance teaching with mobile sensing technology. Pers. Ubiquitous Comput..

[29] Chatzitofis A., Monaghan D., Mitchell E., Honohan F., Zarpalas D., O’Connor N.E., Daras P. (2015). HeartHealth: A cardiovascular disease home-based rehabilitation system. Procedia Comput. Sci..

[30] Mocanu I., Marian C., Rusu L., Arba R. A Kinect based adaptive exergame. Proceedings of the 2016 IEEE 12th International Conference on Intelligent Computer Communication and Processing.

[31] Osgouei R.H., Soulsbv D., Bello F. An Objective Evaluation Method for Rehabilitation Exergames. Proceedings of the 2018 IEEE Games, Entertainment, Media Conference (GEM).

[32] Salvador S., Chan P. (2007). Toward accurate dynamic time warping in linear time and space. Intell. Data Anal..

[33] Müller M. (2007). Information Retrieval for Music and Motion.

[34] Shokoohi-Yekta M., Wang J., Keogh E. On the non-trivial generalization of dynamic time warping to the multi-dimensional case. Proceedings of the 2015 SIAM International Conference on Data Mining.

[35] Lanciault R. Mecanim Humanoids. https://blogs.unity3d.com/2014/05/26/mecanim-humanoids/.

[36] Filkov R. K2-Asset On-line Documentation. https://ratemt.com/k2docs/.

[37] Moon S., Park Y., Ko D.W., Suh I.H. (2016). Multiple kinect sensor fusion for human skeleton tracking using Kalman filtering. Int. J. Adv. Robot. Syst..

[38] Karatsidis A., Bellusci G., Schepers H.M., de Zee M., Andersen M.S., Veltink P.H. (2016). Estimation of ground reaction forces and moments during gait using only inertial motion capture. Sensors.

[39] Guo L., Xiong S. (2017). Accuracy of base of support using an inertial sensor based motion capture system. Sensors.

[40] Reips U.-D., Funke F. (2008). Interval-level measurement with visual analogue scales in Internet-based research: VAS Generator. Behav. Res. Methods.

[41] Shrout P.E., Fleiss J.L. (1979). Intraclass correlations: Uses in assessing rater reliability. Psychol. Bull..

[42] Koo T.K., Li M.Y. (2016). A guideline of selecting and reporting intraclass correlation coefficients for reliability research. J. Chiropr. Med..

[43] Ćwiękała-Lewis K.J., Gallek M., Taylor-Piliae R.E. (2017). The effects of Tai Chi on physical function and well-being among persons with Parkinson’s Disease: A systematic review. J. Bodyw. Mov. Ther..

[44] Sun J., Kanagawa K., Sasaki J., Ooki S., Xu H., Wang L. (2015). Tai chi improves cognitive and physical function in the elderly: A randomized controlled trial. J. Phys. Ther. Sci..

[45] Kottner J., Audigé L., Brorson S., Donner A., Gajewski B.J., Hróbjartsson A., Roberts C., Shoukri M., Streiner D.L. (2011). Guidelines for reporting reliability and agreement studies (GRRAS) were proposed. Int. J. Nurs. Stud..

[46] Kim T., Xiong S. (2017). Comparison of seven fall risk assessment tools in community-dwelling Korean older women. Ergonomics.

[47] Chua P.T., Crivella R., Daly B., Hu N., Schaaf R., Ventura D., Camill T., Hodgins J., Pausch R. Training for physical tasks in virtual environments: Tai Chi. Proceedings of the IEEE Virtual Reality 2003.

[48] Gao Y., Li H., Luo Y. (2015). An empirical study of wearable technology acceptance in healthcare. Ind. Manag. Data Syst..

[49] Kim S.S. (2009). The integrative framework of technology use: An extension and test. MIS Q..

[50] Bhattacherjee A., Hikmet N. Physicians’ resistance toward healthcare information technologies: A dual-factor model. Proceedings of 2007 40th the Annual Hawaii International Conference on System Sciences.

[51] Lee Y., Larsen K.R. (2009). Threat or coping appraisal: Determinants of SMB executives’ decision to adopt anti-malware software. Eur. J. Inf. Syst..

